# A Novel Splicing Mutation in the *ACVRL1/ALK1* Gene as a Cause of HHT2

**DOI:** 10.3390/jcm11113053

**Published:** 2022-05-28

**Authors:** Suriel Errasti Díaz, Mercedes Peñalva, Lucía Recio-Poveda, Susana Vilches, Juan Casado-Vela, Julián Pérez Pérez, Luisa María Botella, Virginia Albiñana, Angel M. Cuesta

**Affiliations:** 1 Departamento Hematología, Instituto Nacional de Enfermedades Neoplásicas, Lima 15038, Peru; suriel_ed@hotmail.com; 2 Departamento Biomedicina Molecular, Centro de Investigaciones Biológicas Margarita Salas (CIB), Consejo Superior de Investigaciones Científicas (CSIC), 280406 Madrid, Spain; mpenalva@ucm.es (M.P.); luciarecio@hotmail.com (L.R.-P.); cibluisa@cib.csic.es (L.M.B.); 3 CIBERER, Unidad 707, Instituto de Salud Carlos III (ISCIII), 28029 Madrid, Spain; 4 Laboratorio Diagnóstico Genético Secugen SL, CIB, CSIC, 28040 Madrid, Spain; s.vilches@secugen.es (S.V.); j.perez@secugen.es (J.P.P.); 5 Facultad de Ciencias Experimentales, Universidad Francisco de Vitoria, Pozuelo, 28223 Madrid, Spain; jucasado@ing.uc3m.es; 6 Departamento Bioingeniería, Escuela Politécnica Superior, Universidad Carlos III de Madrid, 28911 Madrid, Spain; 7 Departamento de Bioquímica y Biología Molecular, Facultad de Farmacia, Universidad Complutense de Madrid, 28040 Madrid, Spain

**Keywords:** *ACVRL1/ALK1*, hereditary hemorrhagic telangiectasia, splicing mutation, Osler-Weber-Rendu disease

## Abstract

Hereditary Hemorrhagic Telangiectasia (HHT) is a rare disorder of vascular development. Common manifestations include epistaxis, telangiectasias and arteriovenous malformations in multiple organs. Different deletions or nonsense mutations have been described in the ENG (HHT1) or ACVRL1/ALK1 (HHT2) genes, all affecting endothelial homeostasis. A novel mutation in *ACVRL1/ALK1* has been identified in a Peruvian family with a clinical history compatible to HHT. Subsequently, 23 DNA samples from oral exchanges (buccal swaps) of the immediate family members were analyzed together with their clinical histories. A routine cDNA PCR followed by comparative DNA sequencing between the founder and another healthy family member showed the presence of the aforementioned specific mutation. The single mutation detected (c.525 + 1G > T) affects the consensus splice junction immediately after exon 4, provokes anomalous splicing and leads to the inclusion of intron IV between exons 4 and 5 in the *ACVRL1/ALK1* mRNA and, therefore, to *ALK1* haploinsufficiency. Complete sequencing determined that 10 of the 25 family members analyzed were affected by the same mutation. Notably, the approach described in this report could be used as a diagnostic technique, easily incorporated in clinical practice in developing countries and easily extrapolated to other patients carrying such a mutation.

## 1. Introduction

Hereditary Hemorrhagic Telangiectasia (HHT), also known as Osler-Weber-Rendu disease, is an autosomal dominant multisystemic vascular dysplasia. Patients with HHT may present multiple symptoms, thus, interfering with efficient clinical diagnosis and optimal patient treatment. Diagnosis is made clinically using the Curaçao criteria: epistaxis (nosebleeds that increase in frequency and quantity with age and are the most common symptom), telangiectases (red-purple spots on the face, lips, mucosa, and fingers), visceral arteriovenous malformations (AVMs) in the lungs, brain, and liver, being the result of abnormal connections between arteries and veins, where the capillary bed has disappeared [1,2,3], and family history. The most severe symptoms include anemia, gastrointestinal bleeding, and complications due to AVMs [3,4]. Typically, if three of the four criteria are present, the patient is diagnosed with HHT [2,5].

Depending on the gene locus affected by mutation, different types of HHT have been reported. The most frequent subtypes, HHT1 and HHT2, are caused by mutations in the *Endoglin (ENG)* and *Activin Receptor-Like Kinase 1 (ACVRL1/ALK1)* genes, respectively, accounting for almost 90% of all HHT cases [6].

HHT3 and HHT4 have been mapped by linkage studies to chromosomal regions 5q31.3-q32 and 7p14, respectively, but no gene has been identified yet [7,8]. Mutations in the *GDF2* gene have been related to HHT-like disease [4,9]. Finally, another gene, *MADH4/SMAD4,* has been identified as being responsible for a combined syndrome, including HHT and juvenile polyposis [10].

A patient with HHT, hereafter the proband, diagnosed by his Health Care Provider in Lima (Peru), according to Curaçao criteria (mentioned above), came for epistaxis treatment to Madrid (Spain). Blood samples were taken from him and from an unaffected relative (with no apparent symptoms) for further genetic analysis. Strikingly, the genetic diagnosis of the HHT patient revealed the presence of a previously unreported mutation, c.525 + 1G > T, affecting the consensus splice site after exon 4 of the *ACVRL1/ALK1* gene. Subsequently, we decided to perform a genetic analysis on all the Peruvian family members who wanted to participate in the study (23 more individuals). For this purpose, buccal swaps were used to obtain the samples in Peru and they were sent to our lab.

In this work, we present the genetic diagnosis, the phenotypic manifestations, of the largest cohort of HHT published so far in Peru. We also demonstrate here, the anomalous presence of the intron IV of *ACVRL1* in the transcripts of the HHT sample, according to the prediction of abnormal splicing due to the mutation present in the family analyzed in this report. Finally, we believe that this is an example of collaboration in the diagnoses of rare diseases, such as HHT, with developing countries that should be emulated.

## 2. Materials and Methods

### 2.1. Human Samples

The entire procedure was approved by the ethical committee of the National Agency of Research in Spain (CSIC), with the reference 075/2017. Blood samples from members of a Peruvian family (a proband meeting HHT Curaçao criteria and a healthy relative) with HHT history were sent from the Hospital Universitario Fundación Alcorcón (HUFA) to our laboratory. Informed consent from both donors was obtained. In addition, 23 buccal swaps samples from direct relatives of the proband (index case) were sent from Peru to our laboratory, with the informed consent of each of the sample donors.

### 2.2. Peripheral Blood Mononuclear Cells (PBMCs) Extraction

A blood gradient was made from the patient’s sample by adding 8 mL of blood on a bed of 4 mL of Ficoll. To achieve separation of the blood components, samples were centrifuged for 20 min at 1000× *g* without brake. Next, the fraction containing the Peripheral Blood Mononuclear Cells (PBMC) was extracted and, together with 5 mL of Phosphate-Buffered Saline (PBS), centrifuged at 1500 rpm for 5 min. The supernatant was removed. The cell pellet was used for two purposes: DNA extraction and cell cultures.

### 2.3. DNA Total Extraction and Sequencing

Total DNA was extracted from Peripheral Blood Mononuclear Cells using the QIAamp Mini Kit (Qiagen, Germany) and following the manufacturer indications.

In the case of buccal swaps DNA extraction was carried out with the same kit, but the first step of lysis was performed with a greater volume as recommended, and lysis was prolonged for 12 h at 56 °C to ensure the highest recovery.

The obtained DNA was subjected to PCR amplification and Sanger sequencing in the case of index case for *ACVRL1/ALK1* and *ENG* exons and intron–exons boundaries, as described in Fernandez-L et al., 2006 [11]. Then, sequences were compared to reference sequences in the databank for *ACVRL1/ALK1* and *ENG*. In the case of direct relatives, exon 4 of *ACVRL1/ALK1* was subjected to PCR amplification using the HotMaster Taq DNA Polymerase (5 Prime, Germany) and subsequent Sanger capillary sequencing [12].

### 2.4. RNA Expression Analysis in Peripheral Blood Mononuclear Cells: RNA Extraction, Retrotranscription and qPCR

Cells isolated after Ficoll gradient centrifugation of 10 mL peripheral blood were plated in single wells of P-6 culture plates and cultured in 2 mL DMEM supplemented with 10% Fetal Bovine Serum (FBS).

After 48 h, adherent cells were detached and centrifuged at 1500 rpm for 5 min. Pellets were subjected to RNA extraction using the NucleoSpin RNA purification kit (Macherey-Nagel GmbH&Co., Düren, Germany), following the manufacturer protocol. Around 1 μg of RNA was retrotranscribed using the Applied Biosystems kit (Thermo Fisher Scientific, Waltham, MA, USA).

Quantitative PCR was performed by FastStart Essential DNA Green Master (Roche, Switzerland) to amplify *β-Actin* and *18S* RNA as housekeeping genes and *ENG* and *ACVRL1/ALK1* as test genes using the primers shown in the following Table 1. Samples were run in triplicate.

## 3. Results

### 3.1. Genetic Analysis of an HHT Family from Peru

The HHT proband was a 62-year-old Peruvian male, diagnosed with HHT by his clinician. The diagnosis fit the Curaçao criteria: nosebleeds, gastric bleeds (AVMs detected by endoscopy), anemia, and family history of nosebleeds. His main problem was anemia, requiring transfusions from time to time due to his severe epistaxis. The patient travelled from Peru to be treated of his epistaxis by sclerotherapy performed by an HHT-ENT referrer at the Hospital Universitario Fundación Alcorcón (HUFA) [13]. In addition, the proband was examined for the presence of AVMs in lung, brain, and liver. No AVMs were found in brain or lung and no hepatic arteriovenous malformations (HAVM) were found. At the same time, blood was extracted for genetic diagnosis. Routine sequencing started with the *ACVRL1/ALK1* gene, due to the absence of pulmonary arteriovenous malformations (PAVMs), which are rare in HHT2, but quite common in HHT1 [14]. Subsequently, *endoglin* was also sequenced in case a mutation might also be present. No changes in the *ENG* sequence were detected.

**Figure 1:**
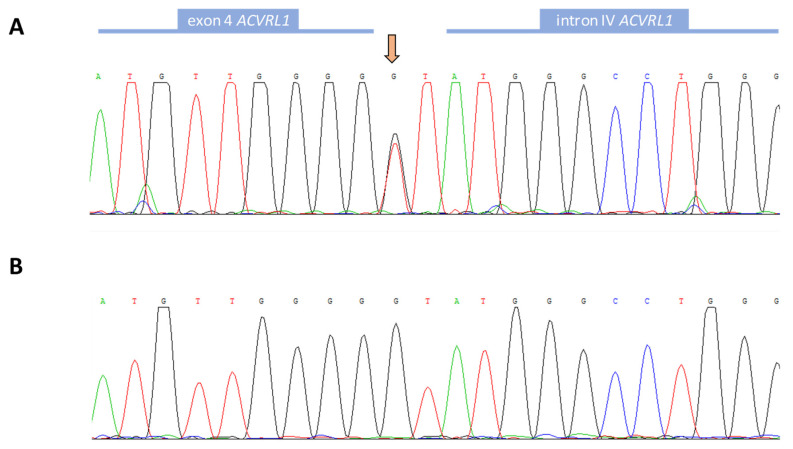
Normal (**B**) and mutated (**A**) exon 4-intron IV boundary sequences of the *ACVRL1/ALK1* gene. These sequences were obtained with a forward primer with the Chromas software.

Genetic analysis found a change in heterozygous condition in *ACVRL1/ALK1*, in the splicing consensus, after exon 4: c.525 + 1G > T (Figure 1), being the first time that this mutation has been described. Figure 1 shows the sequence of the mutated region in the proband (A) and the corresponding wild-type region in a healthy relative (B).

Once the mutation in the index case was known, the family decided to collect samples from all their relatives who intended to be genetically diagnosed. The samples (buccal swaps) were then sent to our lab in Madrid for diagnosis.

**Figure 2:**
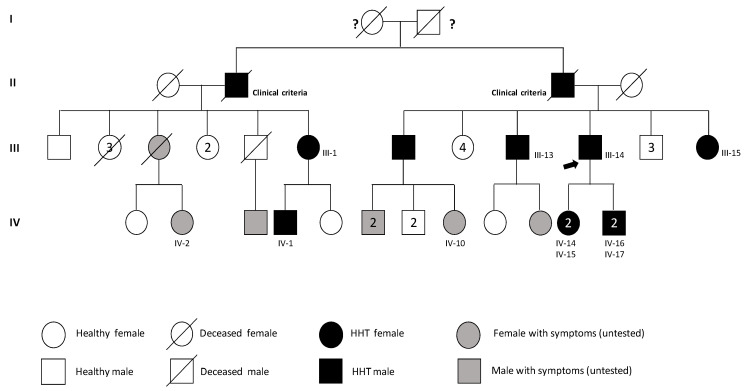
Pedigree of the affected family. In black, individuals with reported HHT (positive for mutation), in open, individuals with no HHT symptoms (negative for mutation) and in grey individuals with HHT clinical symptoms but untested. The black arrow indicates the proband. Numbers inside the symbols indicates the number of siblings with same phenotype. (**I**–**IV** indicate the family generations).

These samples are included in a Peruvian family pedigree showing HHT symptoms (Figure 2). Of these, a total of 24 samples were analyzed for the presence of the mutation found in the index case. Among them, 10 out of the 24 presented the mutation in a heterozygous condition.

### 3.2. Clinical Symptoms

Table 2 shows the clinical symptoms of the positive-for-HHT family members. In general, the symptoms are as expected for HHT type 2, in which the vascular lesions are more related to the gastrointestinal tract [14], as shown by the clinical report of telangiectasias in both the stomach and colon. In two cases, liver screening was performed and revealed the presence of HAVM.

Screening for pulmonary and cerebral AVMs was not performed in most cases. Therefore, we cannot discard the occurrence of PAVMs or cerebral arteriovenous malformations (CAVMs) in some patients, although they would be asymptomatic. Indeed, anemia, a consequence of nosebleeds and of gastrointestinal bleeding, is a symptom present in the older patients.

### 3.3. RNA Expression Levels of ACVRL1 and ENG in Macrophages

**Figure 3:**
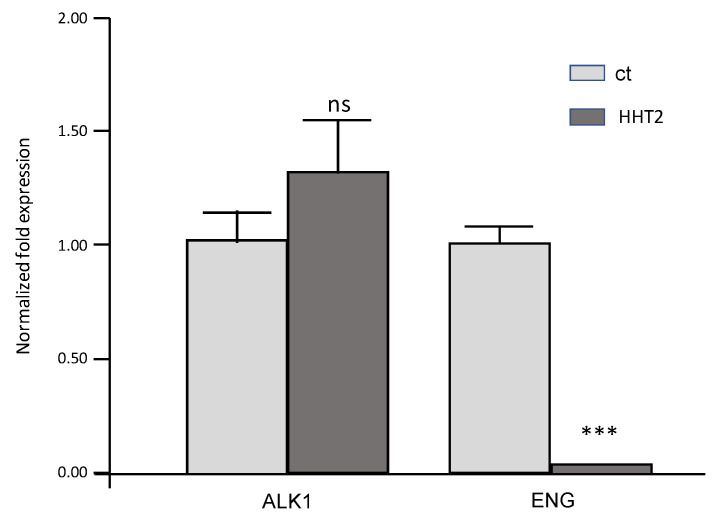
RT-qPCR of ALK1 in the HHT proband (HHT2) and unaffected relative (ct), showing the quantification of ALK1 RNA (ns: *non-significant*; *** *p* < 0.005).

Figure 3 shows the RNA expression of *ACVRL1/ALK1* in the patient compared to a healthy relative. *ENG* expression was also analyzed, since both ENG and ALK1 proteins participate in the TGFβ/BMP9 receptor complex. As described in Section 2.3, RNA expression was analyzed by quantitative PCR in attached cells (PBMCs), mainly macrophages [15,16], which express *ENG* and *ACVRL1/ALK1*. The amount of RNA was normalized to the amount of 18S RNA as a housekeeping gene.

Figure 3 shows the normalized RNA expression levels of *ENG* and *ACVRL1/ALK1* of the proband. While the expression level of *ACVRL1/ALK1* is not significantly different between the proband and healthy control, *ENG* is significantly decreased in the former. It should be stressed that the mutation occurring in *ALK1/ACVRL1* is located at the splice site and, in principle, does not affect the amount of mRNA. On the contrary, the change in *ENG* expression may be a consequence of a transcriptional regulation, in response to decreased functional levels of ALK1.

### 3.4. Isolation of RNA Containing Intron IV of ACVRL1/ALK1 in the Proband

Since the mutation found in this Peruvian family affects the guanine nucleobase belonging to the splicing consensus signal placed at +1 downstream of the exon 4 of *ACVRL1/ALK1*, this change would lead to the inclusion of intron IV in part of the transcripts from the mutated allele in the proband, whereas in the wild-type allele, intron IV would not be present. The presence of the intron would lead to a premature stop codon in translation in half of the transcripts. The stop codon is predicted to be immediately after intron IV, in the first codon of exon 5. Therefore, translation would proceed 48 amino acids within intron IV after exon 4.

**Figure 4:**
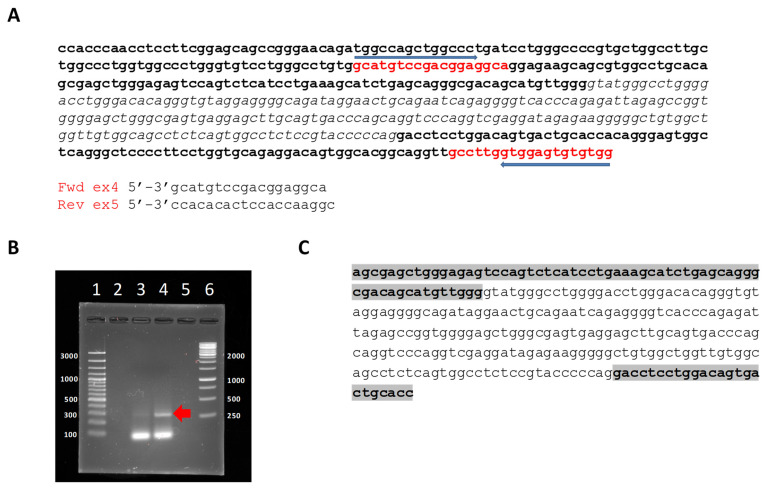
(**A**) DNA sequence of the terminal part of exon 4, intron IV and the beginning of exon 5 of the ACVRL1/ALK1 gene. Sequence of the forward and reverse primers used for PCR amplification show the alignment region within this DNA sequence. (**B**) PCR of the cDNA from HHT and unaffected relative showing, in the case of HHT, the presence of an abnormal band > 250 bp corresponding to the species containing intron IV of ALK1 (highlighted by a red arrow). (**C**) Sequence of exon 4 border and intron IV of ALK1 obtained from the >250 bp band shown in B. Marker sizes are shown.

To demonstrate the presence of an abnormal transcript, total RNA was isolated from the proband and a healthy relative. The RNA was then retrotranscribed and used as a template for PCR amplification using as forward primer that knots within exon 4 but near the exon 4/intron IV boundary, and as reverse, a primer that knots within exon 5 near intron IV of the ACVRL1/ALK1 gene, as shown in Figure 4A. After PCR, the amplified DNA fragments were as shown in Figure 4B. The expected fragment size when intron IV is removed would be 68 bp; however, if intron IV was included (203 bp), another fragment larger than 250 bp would be detected. In Figure 4B, we can see that in lane 2 (healthy control), only a fragment of less than 100 bp is detected. However, in the case of the proband, two fragments could be detected, one of the same size as in lane 2, and one higher up, above the 250 bp size marker. These data conformed to our expectation that the larger fragment represented the RNA with the intron IV incorporated.

To demonstrate that the large fragment contained the intron IV, the DNA fragment was eluted and subjected to Sanger sequencing using the same reverse primer species used for DNA amplification. The sequencing result revealed the presence of the whole intron in this fragment, as shown in Figure 4C.

In conclusion, the evidence reported here strongly suggests that the mutation found is directly responsible for a splicing failure in around half of the transcripts, which will include intron IV of *ACVRL1/ALK1* gene and will generate a premature translation stop in ALK1 synthesis. Thus, the phenotype will be one of haploinsufficiency in those cells expressing ALK1, mainly endothelial cells.

## 4. Discussion

This work presents a new mutation in *ACVRL1/ALK1* as a cause of HHT2, in a large family from Peru, although not all of them have been genetically analyzed. A previous paper [17] described the presence of a novel mutation found in exon 4 of the *ENG* gene c.408delA, at amino acid residue 136, found in four affected members of a family from Peru. However, after this report, and to the best of our knowledge, this manuscript reports the results of the largest cohort of HHT ever published in Peru.

The work is also remarkable, as an example of international, cross-border cooperation, so necessary in rare diseases, where it is difficult to find specialized centers and research on them, especially in areas with fewer resources, as would be the case for HHT and Peru. Taking into account the difficulties in the diagnoses of rare diseases, we turn to clinicians and researchers in developed countries to collaborate and help in the diagnoses, follow-up, and treatments of patients with rare diseases in developing countries. The procedure described here is fast, easy, and feasible.

The cooperation began with the visit to Spain of the index case, diagnosed at the National Institute of Neoplasic diseases in Lima, Peru, as a patient with HHT, following Curaçao criteria. Clinical care included treatment with an ENT specialist in HHT and a clinician of reference in the treatment of epistaxis in HHT, by applying the sclerotherapy technique [13]. Clinical care was also completed with a visit to internal medicine for the detection of possible AVMs in internal organs.

At the same time, genetic diagnosis of the patient was carried out, starting with amplification and Sanger sequencing of the exons and intron–exon boundaries of the *ACVRL1/ALK1* gene, due to the patient’s clinical data. The absence of PAVMs and the presence of nasal, gastric bleeding and HAVMs suggested a higher possibility for having an *ACVRL1/ALK1* mutation. Indeed, a mutation not previously described in the literature was found in c.525 + 1G > T, at the consensus splice site after exon 4. The variant could be classified “a priori” as pathogenic, following the American College of Medical Genetics and Genomics (ACMG) guidelines [18]. In fact, the familial concordance study establishes a strong basis, as individuals III-13, III-14 (proband), and III-15 all had the mutation and met the clinical diagnostic criteria. Moreover, two additional pathogenic variants are known in the same locus (c.525 + 1G > C; c.5252 + 1G > A).

Subsequently, once the bioethics and biosafety measures had been approved, a total of 24 family members residing in Peru underwent genetic analysis. The samples were taken with buccal swaps, which were sent to Spain. Thus, 9 of the 25 relatives were genetically diagnosed as heterozygous carriers of the mutation. The clinical phenotypes were analyzed, within the limitations found due to the dispersion of the affected members in different parts of Peru, and not in the hospital in Lima, where the index case was diagnosed. The clinical symptomatology found makes it desirable that family members with genetic diagnosis of HHT can be followed up by the physician of the National Institute of Neoplasic diseases in Lima, and that a reference unit of HHT for Peru could be created at this hospital.

On the other hand, on the more molecular side, the presence in heterozygosis of this c.525 + 1G > T variant suggested that there would not be correct splicing between exons 4 and 5 of *ACVRL1/ALK1*. A deficiency in the *ACVRL1/ALK1* messenger would not be expected, since in principle, transcription would proceed, although subsequent splicing would be aberrant. Figure 3 shows that there were no significant differences between ALK1 transcript levels in the proband compared to a normal relative. It is also remarkable to see how the amount of *ENG* transcript is downregulated in the proband vs. control. This deficit can be explained because there is a coordinated transcriptional regulation between *ENG* and *ACVRL1/ALK1* described by our group [11,19,20]. Since both ENG and ALK1 proteins are present in the TGF-β/BMP9 signaling complex, when there is a deficit in the ALK1 protein, a negative feedback occurs in ENG transcription, so that at the protein level, the signaling complex is balanced [11,20], thus, adapting to the ALK1 deficit scenario. Different authors in Europe and the United States have made contributions of genotype–phenotype correlations [21,22,23].

As demonstrated in Figure 4, aberrant splicing leads to the inclusion of intron IV in half of the transcripts—those originating from the transcript of the mutated allele. From total RNA isolation, an anomalous RNA species is detected only in the proband. Sequencing of this species confirmed the presence of exons 4 and 5, along with the presence of the intron between them. This aberrant splice variant would result in a premature termination codon, which would not result in a functional protein.

The limitation of this study is the lack of enough biological material, either endothelial cells [19] or macrophages, to perform protein studies to see the amount and type of ALK1 species in both the mutated case and in the healthy family member. In addition, with a primary endothelial cell culture, the amount of surface protein could be analyzed by flow cytometry in comparison with a healthy control. Finally, various experiments, including TGF-β/BMP9 pathway-dependent signaling and functional experiments mentioned above, could be performed.

## 5. Conclusions

- A new mutation in the *ACVRL1/ALK1* gene (HHT2), involving the consensus splice junction immediately after exon 4, c.525 + 1G > T, was found in a member of a large Peruvian family with a history of HHT-compatible symptoms.
- Among 25 members of this family, 10 of them showed the presence of the mutation in a heterozygous condition, while 15 showed the wild-type alleles. The presence of this mutation was correlated with the clinical symptoms of HHT following Curaçao criteria.
- It has been demonstrated that the mutation leads to an abnormal splicing of *ACVRL1/ALK1* intron IV, since an RNA species containing exon4-intron IV-exon 5 was isolated, in addition to normal splicing in around half of the transcripts in an affected member.
- We strongly recommend the screening and follow up of the affected members according to the clinical guidelines of HHT.
- This manuscript may contribute to the awareness of HHT in Peru and to the creation of a reference center together with the patient association, for this rare disease in Peru.

## Figures and Tables

**Table 1:** Primers used for q-PCR assays.

Gene	Fwd 5′-3′	Rev 5′-3′
*18S*	CTAACACGGGAAACCTCAC	CGCTCCACCAACTAAGAACG
*ACVRL1/ALK1*	ATCTGAGCAGGGCGACAGC	GAGGGACACCACGTCAGT
*ENG*	AGCCTCAGCCCCACAAGT	GTCACCTCGTCCCTCTCG
*β-actin*	AGCCTCGCCTTTGCCGA	CTGGTGCCTGGGGCG

**Table 2:** Clinical symptoms of the family members with Curaçao criteria (* indicates index case, F: female, M: male).

Patient	Sex	Age	Epistaxis	Telangiectases	Avms	Screening	Other Diseases
**III-1**	F	64	yes	Face, fingers	----	none	not referred
**III-13**	M	69	yes	Back, face, fingers, legs, tongue,	gastric, colon	gastrointestinal endoscopy, colonoscopy	Gastric metaplasia, Anemia
III-14 *	M	65	yes	Face, fingers, Tongue	gastric, colon, liver	gastrointestinal (endoscopy, colonoscopy), brain, lung, liver,	not referred
**III-15**	M	64	yes	Face, fingers, gums, tongue	gastric	Gastric endoscopy	Parkinson/PVI
**IV-1**	M	38	yes	Not detected	none	not screened	not referred
**IV-2**	F	32	yes	Tongue	none	not screened	not referred
**IV-10**	F	49	yes	Face, fingers, lips, tongue	liver, colon	Colonoscopy, liver	not referred
**IV-14**	F	35	yes	Face, fingers, tongue	none	not screened	not referred
**IV-15**	F	28	yes	Not detected	none	not screened	not referred
**IV-16**	M	26	yes	Chest, face, fingers, tongue	none	not screened	not referred
**IV-17**	M	40	yes	Fingers	none	not screened	not referred

## Data Availability

Data supporting the reported results can be found in our laboratory records. DNA and RNA are part of the collection associated with the research of the group.
